# Revealing the Functional Neuroanatomy of Intrinsic Alertness Using fMRI: Methodological Peculiarities

**DOI:** 10.1371/journal.pone.0025453

**Published:** 2011-09-30

**Authors:** Benjamin Clemens, Mikhail Zvyagintsev, Alexander Sack, Armin Heinecke, Klaus Willmes, Walter Sturm

**Affiliations:** 1 Neurological Clinic, Section Neuropsychology, University Hospital RWTH Aachen, Aachen, Germany; 2 Interdisciplinary Centre for Clinical Research – Neurofunctional Imaging Lab, University Hospital RWTH Aachen, Aachen, Germany; 3 Faculty of Psychology and Neuroscience, Department of Cognitive Neuroscience, Maastricht University, Maastricht, The Netherlands; 4 Neurological Clinic, Clinical Neuropsychology, University Hospital RWTH Aachen, Aachen, Germany; University Medical Center Groningen UMCG, The Netherlands

## Abstract

Clinical observations and neuroimaging data revealed a right-hemisphere fronto-parietal-thalamic-brainstem network for intrinsic alertness, and additional left fronto-parietal activity during phasic alertness. The primary objective of this fMRI study was to map the functional neuroanatomy of intrinsic alertness as precisely as possible in healthy participants, using a novel assessment paradigm already employed in clinical settings. Both the paradigm and the experimental design were optimized to specifically assess intrinsic alertness, while at the same time controlling for sensory-motor processing. The present results suggest that the processing of intrinsic alertness is accompanied by increased activity within the brainstem, thalamus, anterior cingulate gyrus, right insula, and right parietal cortex. Additionally, we found increased activation in the left hemisphere around the middle frontal gyrus (BA 9), the insula, the supplementary motor area, and the cerebellum. Our results further suggest that rather minute aspects of the experimental design may induce aspects of phasic alertness, which in turn might lead to additional brain activation in left-frontal areas not normally involved in intrinsic alertness. Accordingly, left BA 9 activation may be related to co-activation of the phasic alertness network due to the switch between rest and task conditions functioning as an external warning cue triggering the phasic alertness network. Furthermore, activation of the intrinsic alertness network during fixation blocks due to enhanced expectancy shortly before the switch to the task block might, when subtracted from the task block, lead to diminished activation in the typical right hemisphere intrinsic alertness network. Thus, we cautiously suggest that – as a methodological artifact – left frontal activations might show up due to phasic alertness involvement and intrinsic alertness activations might be weakened due to contrasting with fixation blocks, when assessing the functional neuroanatomy of intrinsic alertness with a block design in fMRI studies.

## Introduction

Understanding the concept of attention has repeatedly been a prominent goal in psychological and neuropsychological experimental studies [1], [2]. Attentional research involving human participants provided insights into how perceptual information reaches conscious awareness and enables direct examination of different aspects of brain-behavior relationships [2]–[4]. In most theoretical accounts, attention is not conceptualized as a unitary function, but rather sub-divided into different components representing specific attentional functions. A recent classification of these attentional components suggested by Sturm [5], [6] differentiates between intensity, spatial and selectivity aspects of attention. This classification in turn is partially based on previous theoretical conceptualizations [7]–[9]. In this taxonomy, the selectivity aspects of attention, including focused, selective and divided attention, regulate fast selection of relevant features in complex tasks involving goal-directed control of attention. Furthermore, spatial attention controls for spatial shifts of the attentional focus. The intensity aspects of attention comprise alertness, vigilance and sustained attention, which are usually examined with simple reaction time tasks containing only one target stimulus [5], [10]. In this framework, intensity aspects of attention are of particular interest because they are regarded as a necessary pre-condition for the cognitively more demanding selectivity aspects of attention [6], [11]. Intensity aspects of attention require both bottom-up and top-down control of attentional processing; they regulate basic target detection in simple reaction time tasks without distracting stimuli, and they are influenced predominantly by perceptual intensity, saliency and behavioral relevance of the stimuli [2], [12]–[15]. The taxonomy thus distinguishes between simple, more energetic attentional processing (i.e. intensity aspects) and more complex attentional processing (i.e. selectivity aspects). Moreover, the model by Sturm and colleagues also differentiates between intrinsic and phasic alertness. While the former is considered to be responsible for the internal control of arousal in situations involving non-cued target detection, phasic alertness results in a temporarily increased response readiness due to an external warning cue [6]. Intrinsic alertness involves the internally motivated and controlled maintenance of alertness, whereas phasic alertness can be thought of as an externally triggered activation of the alertness network due to an attention-capturing stimulus presented in the external environment of the participant [10], [16].

Neuropsychological research on intrinsic alertness, involving lesion studies in stroke patients and lateralized stimulus presentation in split-brain patients and healthy volunteers, suggests that the right hemisphere is of crucial importance for this attentional function [17]–[20]. Although multiple studies with patients suffering from right-hemisphere lesions demonstrated increased reaction times during intrinsic alertness, tested with simple reaction time tasks without an external warning cue, different experimental studies revealed that the same patients could still profit from an external warning cue during phasic alertness tasks [19]–[21]. These results have been taken to indicate that only intrinsic-, but not phasic alertness, crucially depends on normal functioning of the right hemisphere [11], [16], [22], [23], [30]. In accordance with this view, and based on additional research in rats with experimentally induced right hemisphere lesions, the following network model of intrinsic alertness was developed by Posner and co-workers: noradrenergic activation, originating from the ponto-mesencephalic part of the brainstem, passes through the thalamus and subsequently projects to the right prefrontal cortex and right parietal cortex [24]–[26]. Furthermore, Mottaghy and colleagues emphasize that the anterior cingulate cortex (ACC) and the right dorsolateral prefrontal cortex (DLPFC) also exert top-.down control during intrinsic alertness, in order to regulate noradrenergic activation originating from the brainstem [23]. The crucial role of the right hemisphere for intrinsic alertness is further supported by the results of recent neuroimaging studies. Increased neuronal activation during intrinsic alertness was reported for parts of the brainstem and the thalamus [27], the anterior cingulate gyrus (ACG), and most consistently for fronto-parietal structures within the right hemisphere [12], [14]–[16], [28]–[30]. Most of these studies involved positron emission tomography (PET) experiments, using a simple block design to investigate the neural correlates of intrinsic alertness. Because the neural network for intrinsic alertness was activated under visual, auditory and somatosensory stimulation, it has been proposed to work in a supra-modal manner. Although the exact localization of key nodes of the intrinsic alertness network can differ slightly among studies, there is general consensus concerning the crucial involvement of the right hemisphere for intrinsic alertness [5], [11], [29]. Recent fMRI studies, aimed at investigating the specific neural correlates of phasic alertness, found increased activation within the left prefrontal or parietal cortex, in addition to intrinsic alertness related areas such as the thalamus, ACG and right fronto-parietal structures [30]–[36]. The presentation of an external cue before the target stimulus in an alertness task thus seems to recruit additional left fronto-parietal brain areas, resulting in a more bilateral activation pattern as compared to intrinsic alertness [5], [30].

Some inconsistencies concerning the functional neuroanatomy of intrinsic and phasic alertness remain. Accordingly, it has been suggested that the relationship between the neural correlates of intrinsic and phasic alertness is vaguely defined and not yet completely understood [4], [35]. Related to this view, a recent study by Pèrin and colleagues reported a right fronto-parietal-thalamic network for both intrinsic and phasic alertness in healthy participants [29]. These results already indicate that the specific neural correlates for intrinsic and phasic alertness might be hard to disentangle completely, due to the similar cognitive nature and the partly overlapping functional neuroanatomy of the two attentional functions. Pèrin and colleagues did not clarify whether the reported network is functionally relevant and specifically related to intrinsic alertness, because their main analysis employed a conjunction approach to find brain areas activated during both types of alertness. Additionally, different experimental conditions, always presented in the same order, were included in one experimental run. This experimental design prevents an unequivocal interpretation of the intrinsic alertness results, because it seems difficult to determine the overall influence which preparatory motor activity, external cueing, order effects, or some form of goal-directed attention (e.g. dividing attention between conditions) might have had on the brain activity measured during the intrinsic alertness condition. Moreover, Pèrin and colleagues reported that the phasic alertness network consists of exactly the same areas (right inferior parietal lobule (IPL), right DLPFC, thalamus, and ACG) as the intrinsic alertness network, although both networks apparently result from a different contrast and different task stages. In light of all of the above, a specific fMRI investigation on the neuronal correlates of the most basal aspect of attentional processing (intrinsic alertness) seems to be well justified. Another reason to further investigate intrinsic alertness is the fact that it has already been proposed that alertness in general is the most neglected and the least understood dimension of attention research to date [4].

With the present study we tried to evaluate the brain activity of a group of healthy volunteers with fMRI, while they were performing an intrinsic alertness task involving non-cued target detection without distracters. By subtraction of an appropriate control task, which requires a comparable amount of sensory-motor control as the intrinsic alertness task itself, we aimed to isolate changes in brain activity specifically related to intrinsic alertness. According to most attentional accounts and results from the literature, one could expect to find a right-lateralized fronto-parietal cortical network, in addition to thalamic and brainstem structures, for intrinsic alertness. But one important question when studying intrinsic alertness seems whether fMRI block designs are generally well suited for studying the functional neuroanatomy of this attentional function, or if the nature of a block design itself inevitably leads to an involvement of the phasic alertness network. It could easily be argued that the repeated switch between task and rather short rest conditions comprises aspects of phasic alerting, with the change between conditions serving as a kind of external warning cue triggering the phasic alertness system. Thus, the primary objective of the present study was to determine the specific neural correlates of intrinsic alertness, if assessed with an fMRI block design experiment. A secondary goal of the present study was to compare our results to previous findings from fMRI studies investigating intrinsic alertness in healthy participants. To this end, we evaluated the brain activity of healthy participants while performing an intrinsic alertness paradigm, which was specifically designed for the purpose of a standardized, diagnostic assessment of this attentional function. First, a block design and straightforward hypothesis-driven statistical analysis were employed, in order to specifically investigate the functional neuroanatomy of intrinsic alertness. Second, we conducted a more fine-grained analysis of the activations obtained during the intrinsic alertness task, focusing more on those parts of the experimental paradigm specifically related to intrinsic alertness. We expected to find increased neuronal activation within the right-hemisphere network for intrinsic alertness, possibly combined with additional left-hemispheric activations due to phasic alertness aspects inherently present in fMRI block designs.

## Methods

### 2.1 Ethics statement

The experimental procedure was approved by the Ethics Committee of the Medical Faculty of the RWTH Aachen University (protocol number: EK 071/09).

### 2.2 Participants

16 healthy volunteers (mean age, 26 years; range 22–36 years; 12 males) with no history of neurological or psychiatric illness participated in the present study. All participants were recruited via public announcement and had normal or corrected to normal vision. The Edinburgh Handedness Inventory [36] was used to determine consistent right-handedness (mean lateralization-quotient = 87; standard deviation, SD = 25). The experimental procedure was approved by the Ethics Committee of the Medical Faculty of the RWTH Aachen University (protocol number: EK 071/09). All participants gave their written informed consent, and everybody received a compensatory payment (25€) for participation in the study. Furthermore, all participants had the same educational level (i.e. obtained the German high-school degree).

### 2.3 Task and procedure

**Figure 1:**
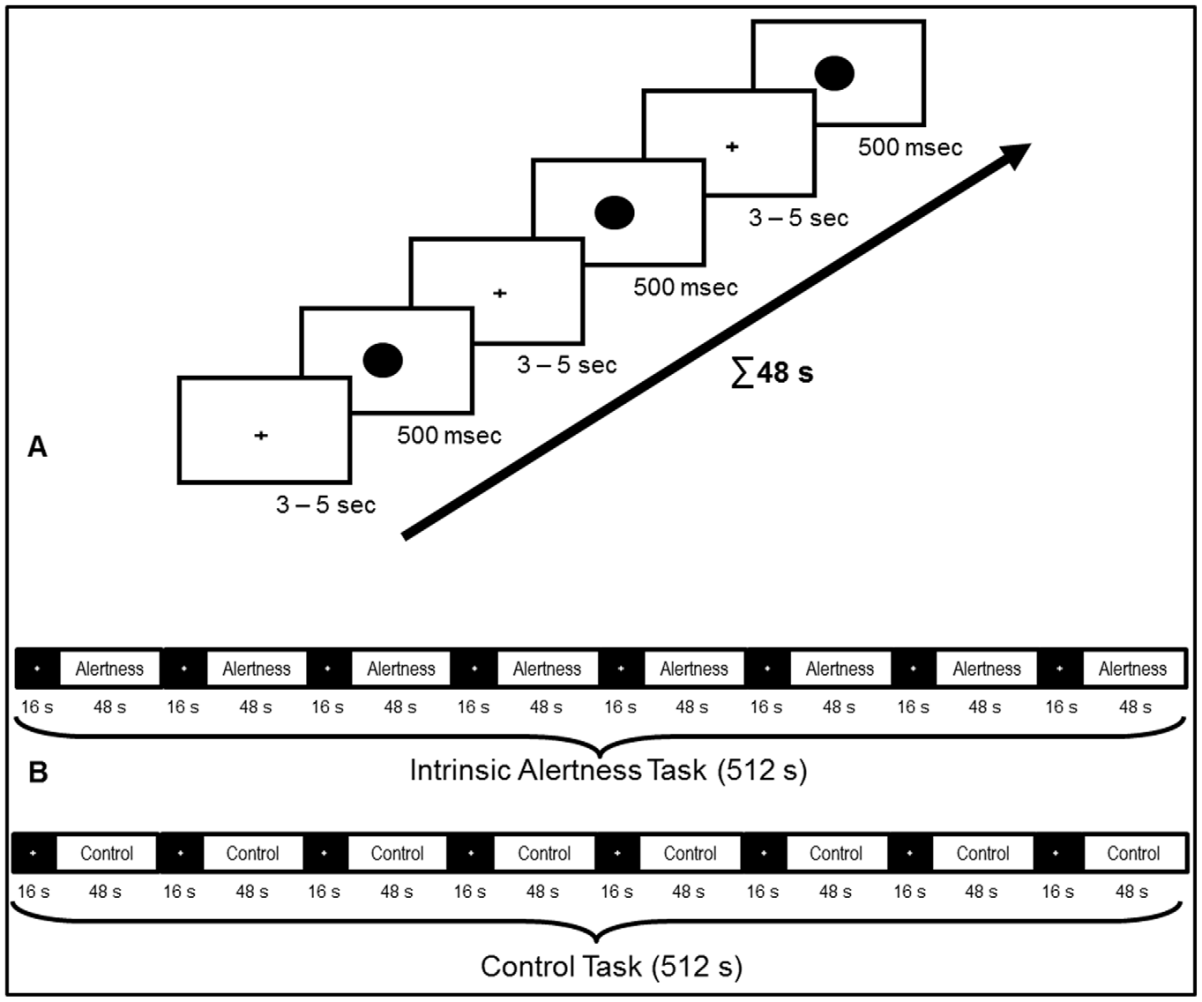
Complete overview of the experimental setup. (**A**) shows the timing of *3 trials* for the intrinsic alertness task. During each trial, participants were instructed to fixate the cross at the center of the screen and wait for the target stimulus, which was a black circle presented at the center of the screen. The inter-stimulus interval was always randomized between 3 and 5 s, and the target stimulus was presented for a maximum duration of 500 ms. This intrinsic alertness task was presented in blocks of 48 s, with a maximum of 14 trials in one block. (**B**) shows the overall timing for both the intrinsic alertness task and the control task, during the whole experimental run. Both tasks were presented in separate experimental runs, and the order was randomized across all participants. Both experimental runs always started with the low-level baseline condition (resting period, 16 s), followed by an experimental block (48 s). Within one experimental run, 8 experimental blocks were alternated with 8 resting periods, resulting in a total duration of 8 minutes and 32 s.

The intrinsic alertness task, which was evaluated for the first time in an fMRI environment, is part of a computerized test-battery to assess sensory and attention functions called WAF (Wahrnehmungs- und Aufmerksamkeitsfunktionen; Schuhfried; Mödling, Austria; 2007). This test-battery contains sub-tests for different attentional functions, such as intrinsic alertness, and is regularly used by psychologists and neuropsychologists to assess attentional deficits in healthy participants or in neurological patients [37]. The intrinsic alertness task chosen was originally designed in our lab (W.S.), to specifically assess intrinsic alertness deficits in stroke patients. Accordingly, much caution was devoted to the technical and methodological aspects of implementing this task within the fMRI environment, in order to optimally preserve its essential features. In a previous behavioral and normative study involving a large sample (*n* = 295) of healthy volunteers, the intrinsic alertness task of the WAF was already validated at the behavioral level, demonstrating good construct and content validity [38]. The results of this behavioral validation study, in combination with the successful application of the paradigm in neurological patients, ensured that the experimental task provides a reliable measure of intrinsic alertness and may thus serve as a good candidate to study the neural mechanisms involved in this attentional function. During the intrinsic alertness task itself, participants were required to press the response button with their right index finger as fast as possible when they detected a target stimulus. The target stimulus was a black circle, 7.25 cm in diameter, presented for 500 ms at the center of the screen (3.6° viewing angle). The inter-stimulus interval for the appearance of the target stimulus was randomized between 3–5 s, and maximally 14 target stimuli were presented in one block. When the target stimulus disappeared, a black central fixation cross was presented to keep participants fixating the center of the screen. Figure 1A illustrates the time course for 3 trials of one experimental block of the intrinsic alertness task. Prior to fMRI measurements all participants underwent a 5 minute training session with verbal feedback, in order to familiarize them with the experimental task before entering the scanner. This training session employed the same task as presented inside the scanner.

To control for the sensory and motor aspects of the task, we introduced a control task which was performed in a separate experimental run. Here, participants watched a static image of the target stimulus presented at the center of the screen. The instruction was to press the response button at a self-paced rate (approximately 1 button press every 2 seconds). Participants were told to press the response button at a self-paced rate and not to count or estimate temporal intervals during the control condition, in order to keep the level of alertness and arousal as low as possible during the control task. Thus, the intrinsic alertness task and the control task shared the same visual stimulus material, required comparable motor output, and differed only with respect to the instructions given to the participant. Brain areas surviving the contrast Intrinsic Alertness Task > Control Task should thus not be activated primarily due to sensory-motor processing or maintenance of a task set. As with the intrinsic alertness task, all participants were familiarized with the control task before performing it in the scanner. Although the control task chosen here paralleled the intrinsic alertness task as much as possible, it is obvious that the intrinsic alertness task still differed from the control task in some aspects of attentional- and sensory-motor processing. Since we asked participants to respond as fast as possible only during the intrinsic alertness task, we nevertheless induced a higher level of cognitive control of alerting during this condition. This should in turn lead to an increased blood oxygenation level - dependent (BOLD) response within areas of the brain relevant for intrinsic alertness.

A block design was employed to study changes in the BOLD signal while participants performed both tasks in direct succession in the scanner. In contrast to event-related fMRI designs, the block design allows the BOLD response to return to baseline between task and resting periods, which is crucial when studying the neural correlates of intrinsic alertness (i.e. the internal *up-regulation* of arousal). To specifically identify brain activation patterns related to intrinsic alertness, we included two different baseline conditions. The high-level baseline condition was the control task described above, and short fixation periods were used as a low-level baseline condition. Both the intrinsic alertness task and the control task were presented in blocks (48 s) and alternated with shorter fixation periods (low-level baseline, lasting 16 s). During those fixation periods which contained a white cross in front of a black screen, participants were instructed to relax and ‘take a break’ from the experimental task. This low-level baseline condition was included to provide enough time for the BOLD signal to return to the baseline-level in brain areas sub-serving intrinsic alertness. We hypothesized that a regulation of intrinsic alertness was not required during those fixation blocks, because the change of the screen color from white to black clearly indicated the beginning of the fixation block and ensured participants that no stimuli were to be expected during resting periods. To keep the influence of expectation and intrinsic control of alertness low during fixation blocks, a change of the screen color from white to black clearly indicated the beginning of the fixation block and ensured participants that when the background color of the screen turned black, no stimuli were to be expected. The efficacy of this procedure was analyzed in a specific evaluation of the time course of BOLD response during the low-level baseline (fixation) condition. Both the intrinsic alertness task and the control task were presented in separate experimental runs. Each participant performed both tasks directly in succession and the order of both tasks was randomized across participants. During one experimental run, 8 activation blocks and 8 fixation blocks were alternated, always starting with a fixation block. An overview of the complete experimental design is depicted in Figure 1B.

### 2.4 Imaging procedures

fMRI measurements were performed at the University Hospital of the RWTH Aachen with a Siemens 3T Trio scanner (Siemens AG; Erlangen, Germany) using a head coil matrix. Each participant underwent two functional runs (intrinsic alertness task+control task) and one anatomical run. During each functional run, 320 functional images were acquired using a spin-echo EPI sequence with the following acquisition parameters: TR = 1600 ms, TE = 30 ms, flip angle = 72°, FOV = 224×224 mm^2^, matrix size = 64×64, 30 transversal slices, slice thickness = 3.5 mm, interleaved scanning acquisition, gap = 0.35 mm. High-resolution anatomical images were acquired for each participant using an MPRAGE sequence with the following acquisition parameters: TR = 2300 ms, TE = 2.98 ms, flip angle = 9°, FOV = 256×256 mm^2^, 176 sagittal slices, slice thickness = 1 mm. The total scanning time for each participant was approximately 35 minutes and the anatomical scan was always performed at the end of the experimental session.′

### 2.5 fMRI data analysis

The brain imaging data were analyzed with the BrainVoyager 2.1 software package (Brain Innovation; Maastricht, The Netherlands). For each functional scan, a time series of 320 images was included. The first 2 volumes of each time series were discarded, to allow the brain to reach a stable magnetized state and to prevent artifacts from transient signal changes at the beginning of each functional run. The functional images were first subjected to linear trend removal, interscan slice time correction using sinc interpolation, temporal high-pass filtering to remove low-frequency drifts of 3 cycles or less, and three-dimensional motion correction using sinc interpolation to correct for small head movements. Functional time series were then spatially smoothed using a 4-mm Gaussian kernel at full-width half-maximum. Subsequently, the functional data sets were transformed into Talairach space [39] by co-registering them with the anatomical scans for each individual participant. A voxel-wise, hypothesis-driven analysis of the BOLD signal, based on the application of the general linear model (GLM) to time series of functional activations [40], [41], was conducted to test for the presence of specific effects related to the different experimental tasks.

For the first part of the analysis, the single-subject GLM of the experiment was computed from the z-normalized volume time courses obtained during the two experimental runs. Each task was presented in a separate run, and separate GLM's were calculated for each task, with the signal values during the 8 activation blocks considered as the effects of interest. To model these effects, one predictor of interest for each task (1 ‘task’ predictor for 8 blocks of 48 s) was defined and entered into the GLM for each participant separately. With the goal of reducing the error variance of the GLM, the following confound predictors were also added to each single-subject GLM: 6 predictors representing the individual motion correction parameters (3 rotational and 3 translational parameters) of each participant, and 2 confound predictors modeling the screen change at the transition between the fixation and the task blocks. These last two predictors (SC1, SC2) were introduced to remove variance potentially related to activation of the phasic alertness system, which was triggered by the change of the background color of the screen from black to white. Thus, the signal values during the first 3.2 s of each activation block and the last 3.2 s of each fixation block were modeled by these 2 confound predictors. Comparable to motion-related variance removed by introducing the individual motion correction parameters as confound predictors, the variance captured by the predictors for the transition between the fixation and the task block was thus removed from the analysis.

For the second part of the analysis, we focused only on the activations during the run containing the intrinsic alertness task, and for this part of the analysis the single-subject GLM was computed from the z-normalized volume time courses obtained during the intrinsic alertness run. Each single-subject GLM thus contained the following predictors: 1 ‘task’ predictor (8 blocks of 48 s), 6 confound predictors representing the individual motion correction parameters (3 rotational and 3 translational parameters). To reveal those activations most specifically related to intrinsic alertness, we included two predictors modeling the beginning (BB) and the end (BE) of each task block. Those predictors covered the first and last 9.6 s (6×1.6 s TR = 9.6 s) of each task block. The predictor at block onset (BB) was included to see how external cueing due to the screen change potentially influenced brain activation patterns. The predictor at the end of the block (BE) was included to see which brain areas were active to solve the task when no screen change had occurred immediately before, when the influence of any external cue was presumably lowest. All predictor time courses, including main and confound predictors for both parts of the fMRI analysis, were derived by convolving an appropriate box-car waveform with a double-gamma hemodynamic response function [42], in order to account for the shape, temporal delay and dispersion of the hemodynamic response. Finally, appropriate dummy predictors, representing those predictors which were not present in a given run, were created and entered into each single-subject GLM.

Subsequently, all 32 single-subject GLM's (16 participants×2 tasks) of the first part of the analysis were entered into a random effects GLM (RFX-GLM), in order to be able to compare the activation during the intrinsic alertness task and the control task at the group level. We hypothesized that the results of the following contrast represent the most specific evaluation of intrinsic alertness: Intrinsic Alertness Task > Control Task. The results obtained by performing a simple subtraction of the low-level baseline condition (fixation block) from the intrinsic alertness condition were most likely still influenced by sensory-motor processing. However, this should influence brain activity during both experimental runs in a comparable manner, because the sensory-motor demands and instructional sets of both tasks were quite comparable. By subtracting the activations during the fixation block and the control task from the activations during the intrinsic alertness task, we hypothesized to provide the most adequate evaluation of the functional neuroanatomy of intrinsic alertness. Thresholding of all statistical maps from the first part of the analysis was performed using an approach based on a three-dimensional extension of the randomization procedure described in [43] for multiple comparison correction. An uncorrected, voxel-level threshold of *p* = 0.05 (*t* = 2.13) was set, and the thresholded maps were then submitted to a whole-brain correction criterion based on the spatial smoothness of the functional data sets and an iterative procedure (Monte Carlo simulation) used to estimate cluster-level false-positive rates. After 10.000 iterations, the minimal cluster-size threshold yielding a cluster-level false-positive rate of 5% was determined to be k = 34 functional voxels. This cluster-size threshold was then applied to the statistical maps, and combined with the voxel-level threshold resulted in an estimated whole-brain corrected *α* = 5% level. In the second part of the analysis, all 16 single-subject GLM's of the intrinsic alertness task were entered into a RFX-GLM, to study the activation patterns during the intrinsic alertness task as precisely as possible. For the second RFX-GLM, the p-values of the resulting statistical maps were thresholded and corrected for multiple comparisons with the false discovery rate approach [44].

For proper visualization, all statistical maps were projected on an optimized, 3D surface reconstruction representing the cortically-aligned group average of the brains of all participants. This reconstruction was derived from the segmented brains of each individual participant. Such a surface-based, cortically driven inter-subject alignment of individual brains was recommended for multi-subject averaging in fMRI experiments investigating cortical structures [41], [45]. First, we used largely automatic segmentation routines [46], to segment the grey/white matter boundary of each individual brain. If necessary, additional manual corrections were applied to improve the results of the segmentation and to ensure that topologically correct mesh representations of all individual brains were created. Subsequently, the individual mesh representations of the 16 brains were ‘averaged’ using the cortex-based alignment procedures of BrainVoyager 2.1 [46], [47]. This way of visualizing functional activations comes with the advantage of being able to report coordinates of activation in standard space (Talairach space), while at the same time being able to show the data on an average brain that represents the optimal alignment of the individual cortical structure of the participants of the present study. As compared to the MNI template brain, which is based on a different and much larger set of participants, the optimized 3D surface reconstruction used here visualizes the cortical structure of all participants with more accuracy. All coordinates of activation are reported in the coordinate system of Talairach and Tournoux [39].

Averaged time course plots were created for several brain areas of interest, which were activated in different contrasts from both parts of the fMRI analysis. Linear de-trending was performed on all plots shown in the present study. For the first part of the analysis, averaged time course plots depict the BOLD response during both experimental runs, presented as percent signal change relative to the low-level baseline (fixation), and averaged across the 8 task blocks (8×48 s). For both curves, time zero corresponds to the beginning of the first block of the experimental task (intrinsic alertness task or control task). Functional activation clusters derived from the contrast Intrinsic Alertness Task > Control Task served as seed regions for the averaged time course plots, visualizing – for both tasks separately – the mean BOLD response of all participants for one brain area of interest. Averaged time course plots were calculated for the voxels within a sphere of 5 mm diameter, centered around the peak voxel of the functional activation clusters resulting from the contrast Intrinsic Alertness Task > Control Task from the RFX-GLM of the first part of the analysis. For calculation of the baseline condition, all 10 time points (10×1.6 s = 16 s) of the 8 fixation blocks were averaged. These plots provide an illustrative way to check whether the mean BOLD response of a specific brain area, averaged across all participants and over the complete duration of the task, was consistently increased during intrinsic alertness as compared to the control task. For the second part of the analysis, averaged time course plots, explicitly showing the evolution of the BOLD response during the low-level baseline condition (fixation), were calculated. In this case, the plots depict the BOLD response presented as percent signal change relative to the last three time points (3×1.6 s = 4.8 s) of each intrinsic alertness block. The plots were calculated for all voxels within a sphere of 5 mm diameter, centered around the peak voxel of some of the functional activation clusters resulting from the contrast Block-Ending > Block-Beginning, resulting from the RFX-GLM of the second part of the analysis. These plots thus visualize the BOLD response, averaged across all participants and across the 8 fixation blocks (8×16 s), in several intrinsic alertness-related areas and in 2 brain areas not typically involved in processing intrinsic alertness. This part of the analysis should help to verify whether the change of the screen color between task and fixation conditions effectively prevented increased activity within the intrinsic alertness network already during the fixation block.

### 2.6 Behavioral data analysis

Due to technical problems, both the reaction times (RT) and the number of key presses of two participants were not recorded. The first step in the behavioral data analysis was to average RT for all trials contained in one activation block, which was done separately for each of the 8 task blocks. Subsequently, we calculated the mean RT of the 14 participants, averaged across all 8 task blocks of one experimental run. A one-way repeated measures ANOVA was conducted in order to test the mean RT of the 8 experimental blocks (averaged across 14 participants) for presence of statistically significant differences. A one -sample t test was used to check whether the mean number of key presses - averaged across 14 participants and over the 8 blocks - during the control task was significantly different from the mean number of key presses made during the intrinsic alertness task.

## Results

### 3.1 Behavioral results

We first checked that the mean RT for all 14 participants (range = 210 ms–324 ms) lay within the range of RT considered appropriate for young participants performing the diagnostic assessment version of the intrinsic alertness task [38]. The mean RT averaged across all participants (259 ms; SD = 30.5 ms) in fact represented a performance level normally reported for young participants [38]. The one-way repeated-measures ANOVA, including the RT of 14 participants, with the 8 experimental blocks as within-subject factors, revealed no significant differences between the mean RT of the 8 experimental blocks F(5, 67) = 1.548, *p* = 0.185. This result indicates a constant level of behavioral performance across all experimental blocks, which justified the process of averaging brain activity of each participant across the duration of the whole experimental run. No omission errors were recorded during the intrinsic alertness task performed inside the MR scanner, indicating that the task was accurately performed by all participants. Concerning the number of key presses, averaged across 14 participants and over the 8 blocks of the intrinsic alertness and the control task, the one-sample t test revealed a significantly higher mean number of key presses in the control condition (344; SD = 255) as compared to the number (103) of key presses to be made in the alertness condition; t(13) = 3.52, *p* = 0.004.

### 3.2 fMRI results

**Figure 2:**
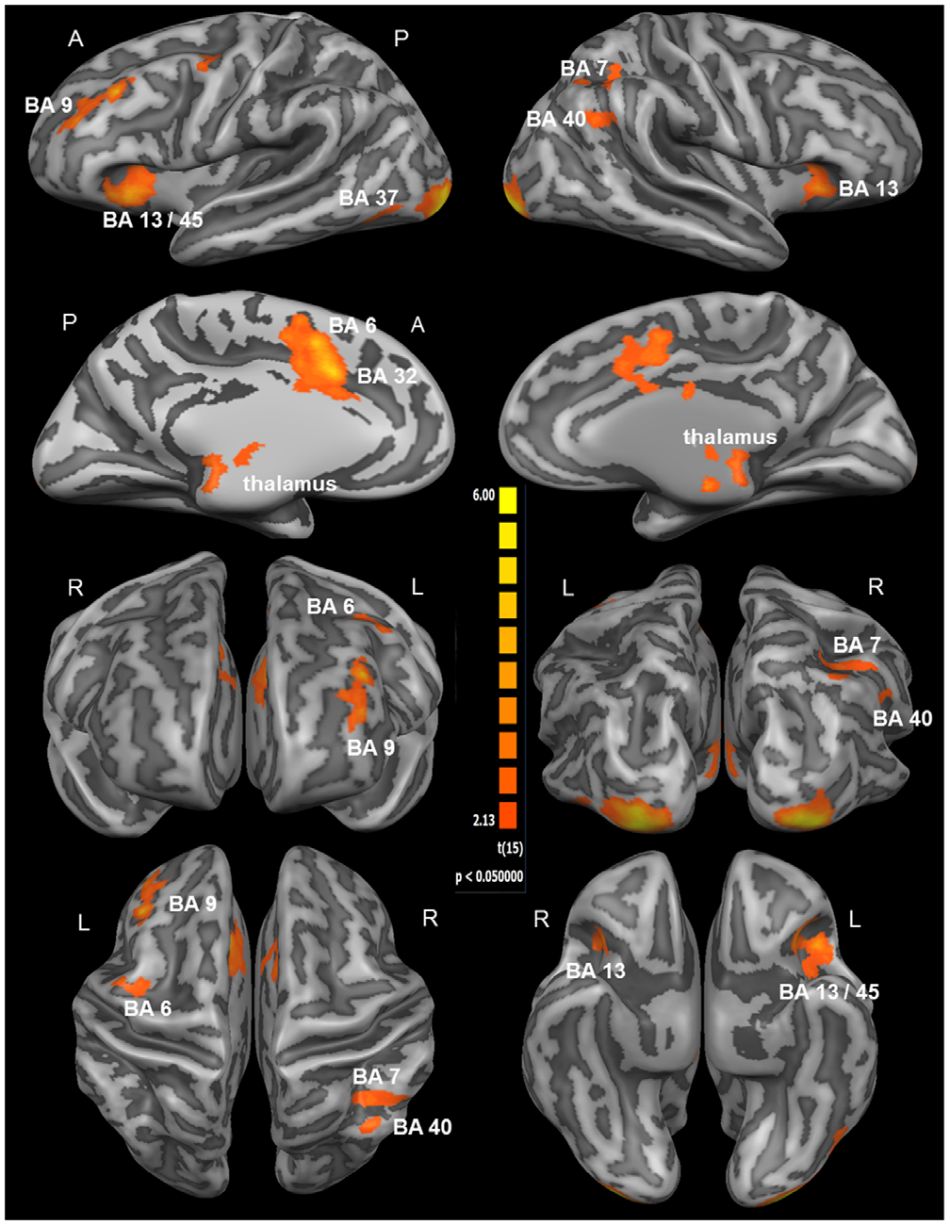
Whole-brain results of the first part of the fMRI analysis (RFX-GLM; *n* = 16). The displayed clusters of activation result from the contrast Intrinsic Alertness Task > Control Task, showing all cortical activations related to the processing of intrinsic alertness. For visualization, results were projected onto the optimized 3D surface reconstruction which represents the average brain of all participants. At the individual voxel-level, activations were thresholded at *p* = 0.05 (uncorrected). Subsequently, a cluster-size threshold of k = 34 voxels was applied, which together resulted in a cluster-level false-positive rate of 5% (whole-brain corrected *p* = 0.05). The first two rows show lateral and medial views of the brain, the third row shows the front and back view of the brain, and the fourth row depicts top and bottom view of the brain. For a complete list of functional activations resulting from the contrast Intrinsic Alertness Task > Control Task, see Table 1. *(A = anterior; BA = Brodmann area; P = posterior; R = right hemisphere; L = left hemisphere)*.

**Figure 3:**
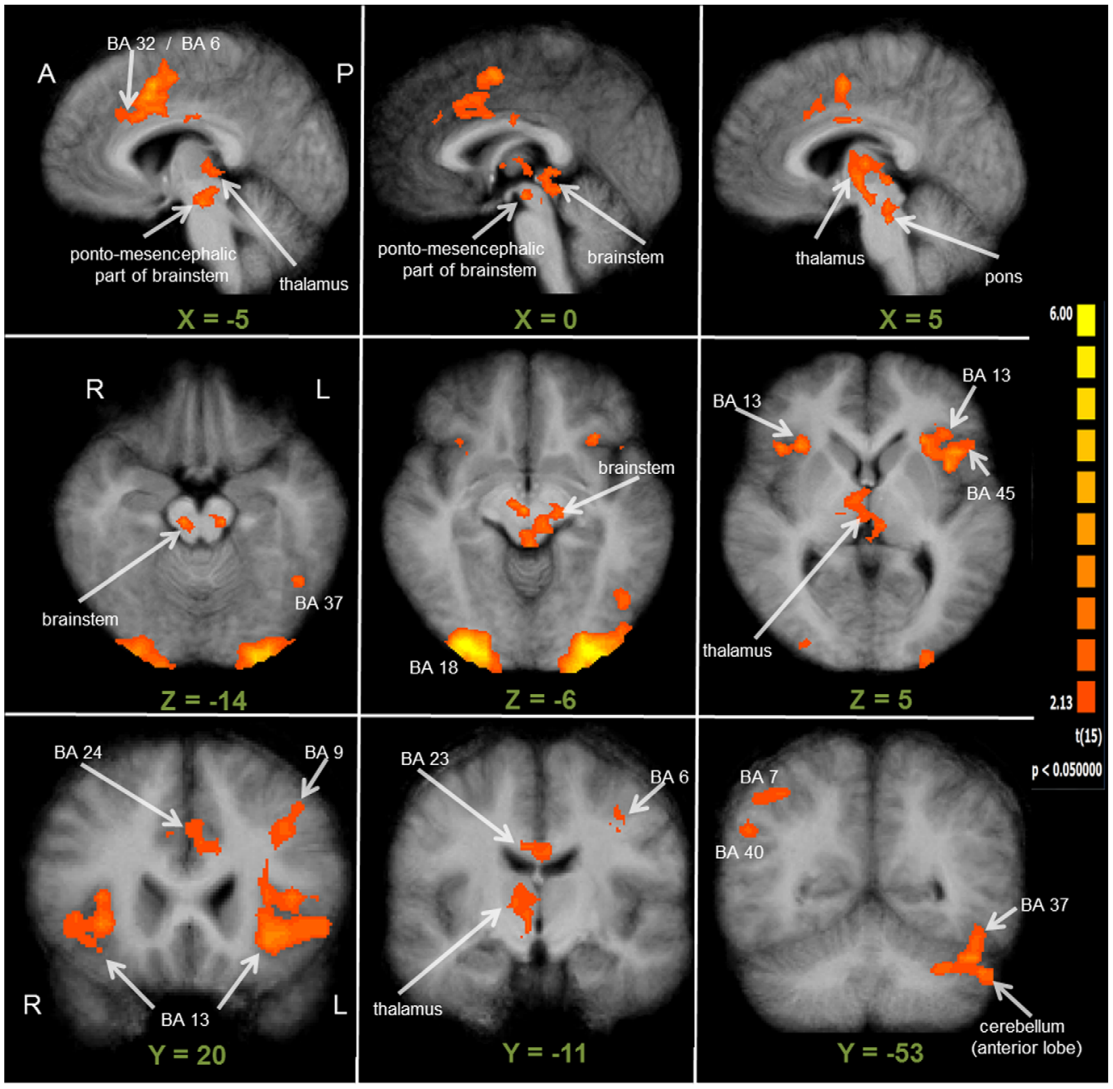
Whole-brain results of the first part of the fMRI analysis (RFX-GLM; *n* = 16). All clusters of activation displayed here are derived from the contrast Intrinsic Alertness Task > Control Task, depicting the same contrast as in Figure 2. For better visualization, especially for subcortical and thalamic structures, this figure shows the results being projected onto the average brain of all 16 participants. At the individual voxel-level, significant activations were thresholded at *p* = 0.05 (uncorrected). Additionally, a cluster-size threshold of k = 34 voxels was applied, which together resulted in a cluster-level false-positive rate of 5% (whole-brain corrected *p* = 0.05). The first row depicts sagittal slices, covering all activations located close to the midline and the medial part of the brain. Transversal slices, covering the brainstem and the thalamus, are shown in the second row. The third row depicts three coronal slices, illustrating the frontal, parietal and thalamic activations, and the activation within the cerebellum, extending into the fusiform gyrus (BA 37). All results are shown in radiological convention. *(A = anterior; BA = Brodmann area; P = posterior; R = right hemisphere; L = left hemisphere)*.

Group activation maps for the contrast Intrinsic Alertness Task > Control Task, with significant clusters of activation projected onto the average brain of all participants, are depicted in Figures 2 and 3. The set of brain areas revealed by this contrast showed increased activation during the intrinsic alertness task as compared to the control task. In the right hemisphere, significant clusters of activation were localized in the anterior part of the insula (BA 13), the inferior and superior parietal lobule (BA 7/40), and in the inferior occipital gyrus (BA 17/18). The largest and strongest cluster of activation within the right hemisphere (see Table 1) was located around the inferior occipital gyrus, spanning both primary and secondary visual cortices (V1/V2). For the left hemisphere, significant clusters of activation were found in the middle frontal gyrus (BA 9), the anterior part of the insula (BA 13), the lateral portion of the supplementary motor area (SMA) in BA 6, and the inferior occipital gyrus (BA 18). Only in the left hemisphere, the activation within the insula extended further towards the pars triangularis, covering BA 13 as well as BA 45. The strongest cluster of activation within the left hemisphere was found within the inferior occipital gyrus, spanning both V1 and V2. Activations in the medial parts of the brain were located around the anterior part of the cingulate gyrus (BA 24/32) and the medial part of the SMA (BA 6). As depicted in Figure 3, the ACG activation also included a more posterior part of the cingulate gyrus (BA 23), although the peak coordinates for this cluster (see Table 1) were clearly located within the dorsal portion of the ACG. Besides the clusters within the right and left occipital cortex, the ACG/SMA cluster exhibited the largest region size and the smallest p-value of all activated clusters (see Table 1).

**Table 1:** List of fMRI activations for the contrast Intrinsic Alertness Task > Control Task.

Anatomical Region	BA	x	y	z	t - statistic	p - value	No. of voxels
*(Intrinsic Alertness Task > Control Task)*							
L middle frontal gyrus	9	−34	22	36	5.79	0.000035	2626
R insula	13	32	19	3	4.37	0.000537	1503
L insula/inferior frontal gyrus	13/45	−43	16	−1	5.23	0.000099	5364
L anterior cingulate gyrus/supplementary motor area	6/24	−10	13	33	6.65	0.000008	10836
L supplementary motor area	6	−37	−5	45	3.89	0.001455	2407
/ponto-mesencephalic part of brainstem		−10	−23	−12	4.82	0.000219	4139
R thalamus (medial dorsal nucleus)		3	−14	9	3.42	0.003838	640
R inferior parietal lobule	7/40	29	−27	36	3.54	0.002986	2625
L cerebellum (extending into BA 37)	37	−43	−53	−24	4.22	0.000726	3092
L inferior occipital gyrus	17/18	−31	−92	−9	8.49	<0.000001	7982
R inferior occipital gyrus	17/18	29	−89	−6	6.76	0.000006	5818

All x, y, and z values given here represent stereotaxic coordinates according to the coordinate system by Talairach and Tournoux (1988). The statistical values in the next columns correspond to the *t*-statistics and the *p*-values of the activation maxima (peak voxel) within each anatomical region. All activations were thresholded at *p* = 0.05 (uncorrected), which together with the cluster-size threshold of k = 34 voxels, resulted in an overall cluster-level false-positive rate of 5% (whole-brain corrected *p* = 0.05). *(BA = Brodmann area; R = right hemisphere; L = left hemisphere)*.

Concerning sub-cortical structures, significantly activated clusters were found in the ponto-mesencephalic part of the brainstem possibly involving the locus coeruleus, the thalamus (medial dorsal nucleus) and the anterior lobe of the left cerebellum. Although the cluster in the left cerebellum extended further dorsally into the fusiform gyrus (BA 37), the peak coordinates for this cluster (see Table 1) were located well within the anterior lobe of the cerebellum. The clusters of activation within the brainstem and the thalamus appeared to be directly connected to each other (see Figure 3), and covered parts of the brainstem, the pons, and the medial dorsal thalamic nucleus. Table 1 provides a complete list of functional activations resulting from the contrast Intrinsic Alertness Task > Control Task. In order to assess the consistency of those results, we additionally verified that a comparable set of brain areas was also activated during the contrast Intrinsic Alertness Task > Fixation. Results of this contrast (not shown here) revealed almost no additional activations as compared to the Intrinsic Alertness Task > Control Task contrast. The only exception in the Intrinsic Alertness Task > Fixation contrast was a significant cluster of activity at the dorsal portion of the central sulcus (BA 4), which was very likely related to the motor output involved in the intrinsic alertness task. All other activations resulting from the Intrinsic Alertness Task > Control Task contrast were also revealed by the Intrinsic Alertness Task > Fixation contrast, but functional activations resulting from the Intrinsic Alertness Task > Fixation contrast showed lower *p* values and were spatially more extended. Thus, a comparable bilateral fronto-parietal-cingulate-thalamic-brainstem network was also revealed by the Intrinsic Alertness Task > Fixation contrast.

**Figure 4:**
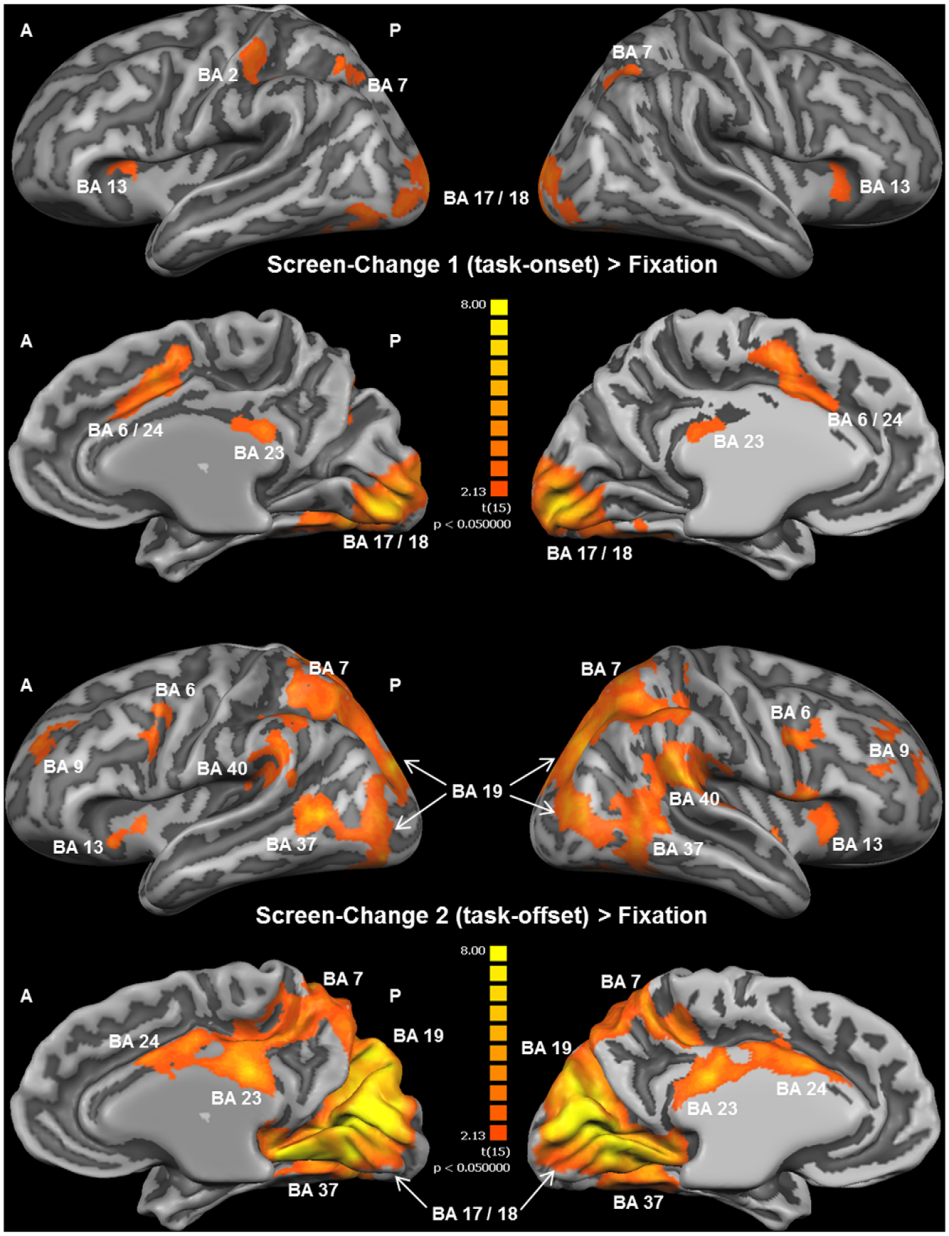
Whole-brain results for the block-onset and block-offset predictors (RFX-GLM; *n* = 16). The displayed clusters of activation result from the contrast SC 1 > Fixation ( = block-onset) in the upper part of the Figure, and from the contrast SC 2 > Fixation ( = block-offset) in the lower part of the Figure. For visualization, results were projected onto the optimized 3D surface reconstruction which represents the average brain of all participants. At the individual voxel-level, significant activations were thresholded at *p* = 0.05 (uncorrected). Additionally, a cluster-size threshold of k = 34 voxels was applied, which together resulted in a cluster-level false-positive rate of 5% (whole-brain corrected *p* = 0.05). As illustrated here, functional activations were spatially more extended and bilateral in the SC 2 > Fixation contrast, as compared to the SC 2 > Fixation contrast. A detailed list of functional activations resulting from both contrasts shown in this figure can be found in Table 3. *(A = anterior; BA = Brodmann area; P = posterior)*.

The brain areas significantly activated during the time periods captured by the two predictors modeling the screen change at the onset (SC 1) and the offset (SC 2) of the task blocks are depicted in Figure 4. The figure shows the contrasts SC 1 > Fixation and SC 2 > Fixation, and it can be seen that the number of activated brain areas, along with the corresponding activation levels, for the SC 2 predictor was generally higher as compared to the activations obtained for the SC 1 predictor. For both the SC 1 and the SC 2 predictor, increased activation was found bilaterally at the primary visual cortices (V1/V2), the superior parietal lobule, the anterior and posterior cingulate gyrus, and the insular cortex. Beyond these common activations, the SC 1 predictor revealed increased activation only around the left post-central gyrus (BA 2). Specifically for the SC 2 predictor, increased activation was observed bilaterally at the intersection of the dorsal portion of the superior temporal gyrus and the inferior parietal lobule (BA 40), the fusiform gyrus (BA 37), the SMA, and the frontal cortices. As illustrated in Figure 4, the visual and cingulate activations were spatially more extended in the SC 2 > Fixation contrast. And compared to the SC 1 > Fixation contrast, the pattern of activity was generally more bilateral in the SC 2 > Fixation contrast, now covering also right IPL and DLPFC. For a detailed list of activated brain areas during the contrasts SC 1 > Fixation and SC 2 > Fixation, see Table 2.

**Table 2:** fMRI results for the contrasts showing block-onset and block-offset predictors.

Anatomical Region	BA	x	y	z	t - statistic	p - value	No. of voxels
*(SC 1 > Fixation)*							
R insula	13	30	20	5	2.7	0.016342	555
L insula	13	−29	18	8	3.01	0.008542	843
/anterior cingulate gyrus/precentral gyrus	6/24	−9	6	44	3.97	0.001049	8102
L post-central gyrus	2	−41	−28	42	3.6	0.002620	3154
/posterior cingulate gyrus	23	2	−31	24	3.56	0.002855	1152
L superior parietal lobule	7	−27	−64	40	3.45	0.003581	1990
R superior parietal lobule	7	27	−61	39	3.95	0.001295	2239
L & R inferior occipital gyrus/lingual gyrus	17/18	−9	−84	−4	6.5	<0.00001	>9999
*(SC 2 > Fixation)*							
L middle/superior frontal gyrus	9	−23	42	28	4.58	0.000361	1549
R middle/superior frontal gyrus	9	23	38	33	6.34	0.000013	4123
/anterior cingulate gyrus	24	−2	12	30	5.49	0.000062	3123
R insula	13	38	8	5	6.1	0.000019	4864
L insula	13	−37	7	0	4.76	0.000241	1332
R precentral gyrus	6	35	2	31	4.74	0.000264	2280
L precentral gyrus	6	−34	−3	42	4.65	0.000306	1478
/posterior cingulate gyrus	23	0	−29	27	7.3	0.000003	8439
R inferior parietal lobule	40	53	−37	31	5.98	0.000025	5816
L inferior parietal lobule	40	−54	−41	32	4.35	0.000572	2955
L superior parietal lobule	7	−36	−56	49	6.97	0.000004	5341
R superior parietal lobule	7	33	−56	47	7.27	0.000003	7256
R fusiform gyrus	37	−47	−54	1	6.01	0.000024	3336
L fusiform gyrus	37	−48	−57	−12	7.62	0.000002	5581
L & R inferior occipital gyrus/lingual gyrus	17–19	−4	−78	6	10.25	<0.00001	>9999

All x, y, and z values given here represent stereotaxic coordinates according to the coordinate system by Talairach and Tournoux (1988). The statistical values in the next columns correspond to the *t*-statistics and the *p*-values of the activation maxima (peak voxel) within each anatomical region. All activations were thresholded at *p* = 0.05 (uncorrected), which together with the cluster-size threshold of k = 34 voxels, resulted in an overall cluster-level false-positive rate of 5% (whole-brain corrected *p* = 0.05). *(BA = Brodmann area; R = right hemisphere; L = left hemisphere)*.

**Figure 5:**
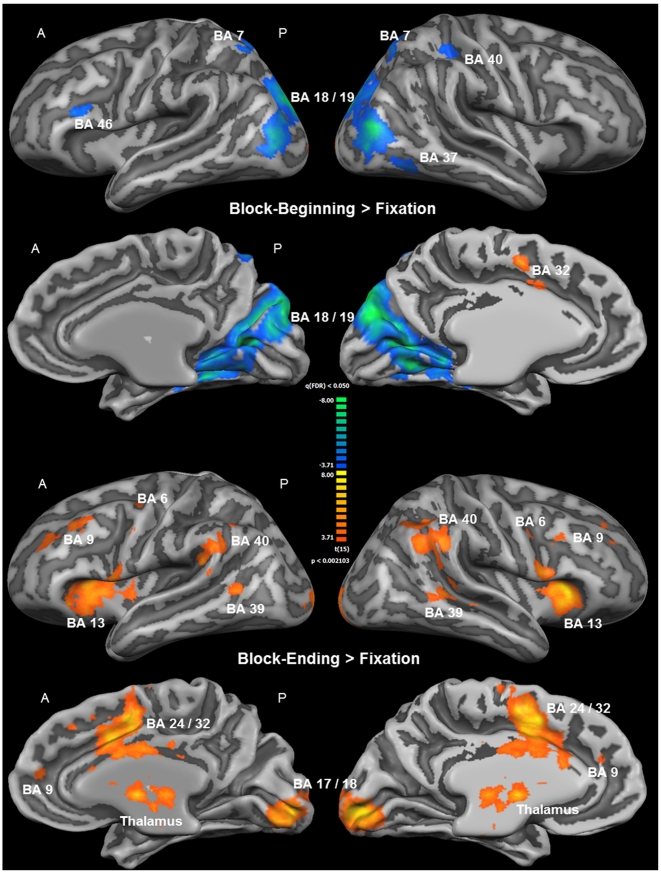
Whole-brain results of the second part of the fMRI analysis (RFX-GLM; *n* = 16). The displayed clusters of activation result from the contrast Block-Beginning > Fixation in the upper part of the Figure, and from the contrast Block-Ending > Fixation in the lower part of the Figure. For visualization, results were projected onto the optimized 3D surface reconstruction which represents the average brain of all participants. All activations were thresholded at q(FDR) < 0.05 (t = 3.71). The beginning of the intrinsic alertness task is associated with less activation, relative to the low-level baseline (color code, blue to green), including alertness-related areas such as the right parietal cortex, and the only brain area showing increased activation (color code, orange to yellow) during this stage of the task is the ACC. The ending of the intrinsic alertness task, on the other hand, is associated with widespread activations, including the fronto-parietal intrinsic alertness network, and decreased activity was not found as a result from this contrast. For a complete list of functional activations resulting from the contrasts Block-Beginning > Fixation and Block-Ending > Fixation, see Table 2. *(A = anterior; BA = Brodmann area; P = posterior)*.

**Figure 6:**
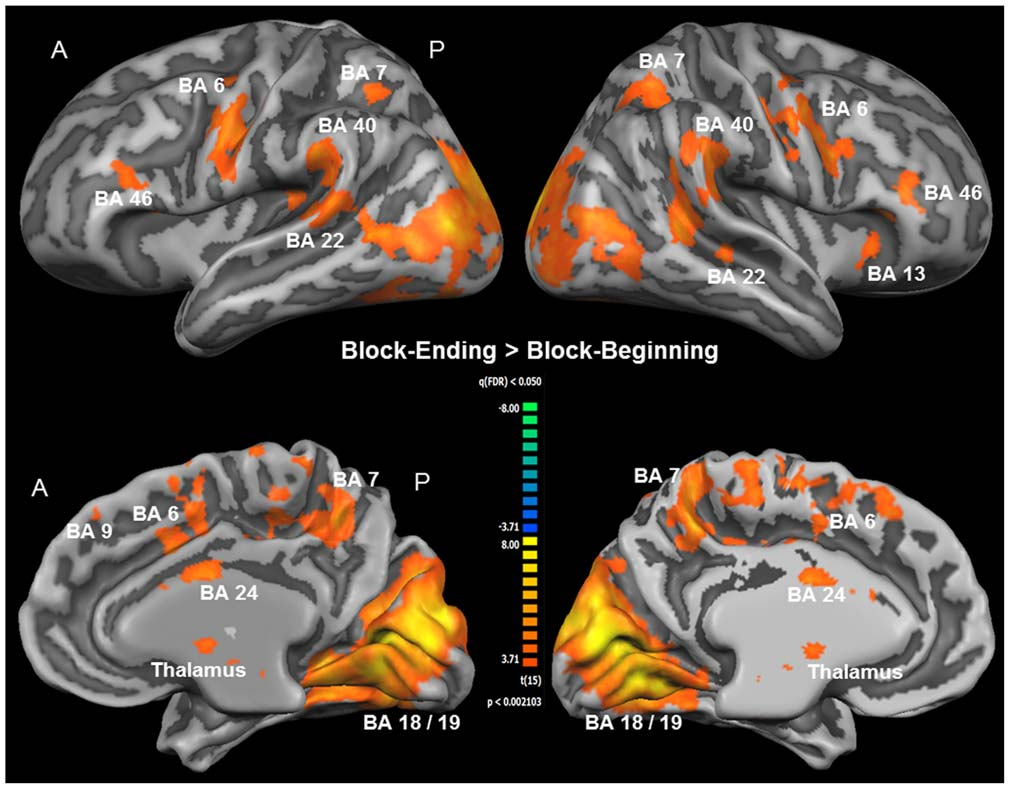
Whole-brain results of the second part of the fMRI analysis (RFX-GLM; *n* = 16). All clusters of activation shown here are the result of the contrast Block-Ending > Block-Beginning, specifically representing how the intrinsic alertness task was processed in the absence of any potential external cue, such as the change of the screen color happening at the beginning of the task block. For visualization, results were projected onto the optimized 3D surface reconstruction, representing the average brain of all participants. All activations were thresholded at q(FDR) <0.05 (t = 3.71). Brain areas showing higher activation during the ending than during the beginning of the intrinsic alertness task included the right and left fronto-parietal cortices, the thalamus and the ACC, among others. For a complete list of functional activations resulting from the contrast Block-Ending > Block-Beginning see Table 3. *(A = anterior; BA = Brodmann area; P = posterior)*.

Group activation maps from the second part of the analysis revealed different activation patterns during the beginning and the end of the intrinsic alertness task block. While activation was not widely increased, and in many parts of the brain even decreased during the beginning of each task block, a widespread increase of activation was observed at the end of each task block (see Figure 5). Table 3 contains a complete list of functional activations resulting from the contrasts Block-Beginning > Fixation and Block-Ending > Fixation. The only area showing increased activation during the first part of the task was the dorsal ACC, while less activation, relative to the low-level baseline, during the first part of the task was observed bilaterally in visual and parietal areas, and in the left BA 46. For the last part of the task, increased activation was found within several intrinsic alertness related areas, such as the thalamus, dorsal ACG, DLPFC, IPL, and additionally in the insula, the SMA, and the occipital cortex (see Figure 5). All of the activations resulting from the contrast Block-Ending > Fixation were bilateral in nature, and less activation, relative to the low-level baseline, was found as a result of this contrast. Directly comparing which areas were more activated during the end of the block, as compared to the beginning of the block, should enable us to find out which brain areas sub-serve intrinsic alertness when the influence of any potential external cueing (e.g. screen change) was presumably lowest. The corresponding contrast Block-Ending > Block-Beginning (see Figure 6 and Table 4) revealed increased activation also within the fronto-parietal-thalamic-cingulate network, comparable to the results of the contrast Block-Ending > Fixation. Increased activations were also found within the occipital cortex, the SMA, the SPL bilaterally, and the right insula. To sum up, the second part of the analysis showed that increased activation within the intrinsic alertness network was only found when focusing on the end of the experimental task.

**Table 3:** fMRI results for the Block-Beginning > Fixation/Block-Ending > Fixation contrasts.

Anatomical Region	BA	x	y	z	t - statistic	p - value	No. of voxels
*(Block-Beginning > Fixation)*							
L middle frontal gyrus	46	−40	19	21	−8.01	0.000001	961
R inferior parietal lobule	40	32	−38	39	−5.3	0.000087	548
R superior parietal lobule	7	29	−50	48	−4.81	0.000231	494
R fusiform gyrus	37	46	−55	−10	−5.01	0.000151	1180
L superior parietal lobule	7	−24	−62	53	−4.86	0.000209	463
L & R inferior occipital gyrus	18/19	11	−83	27	−11.47	<0.000001	>9999
/anterior cingulate gyrus	24/32	−1	7	45	7.39	0.000002	1521
*(Block-Ending > Fixation)*							
/medial frontal gyrus	9	−1	38	27	5.46	0.000064	624
R superior frontal gyrus	9	20	37	33	5.37	0.000078	568
L middle frontal gyrus	9	−28	28	32	5.77	0.000036	1950
R insula	13	29	19	16	9.01	<0.000001	6555
L insula	13	−34	16	15	9.65	<0.000001	7675
/anterior cingulate gyrus/precentral gyrus	6/24	−9	7	45	8.86	<0.000001	>9999
/thalamus (anterior nucleus)		−4	−2	6	7.39	0.000002	2976
L precentral gyrus	6	−48	−4	35	6.34	0.000013	671
R precentral gyrus	6	41	−8	36	4.99	0.000162	223
/thalamus (pulvinar)		−7	−26	9	7.9	0.000001	2684
R supramarginal gyrus/inferior parietal lobule	40	53	−44	33	6.04	0.000023	2630
L supramarginal gyrus/inferior parietal lobule	40	−53	−45	−29	7.63	0.000002	1503
R superior temporal gyrus	39	50	−50	6	5.92	0.000027	1102
/cerebellum (anterior lobe, culmen)		−1	−53	−9	6.4	0.000012	2528
L superior temporal gyrus	39	−49	−56	6	5.49	0.000061	396
L & R inferior occipital gyrus/lingual gyrus	17/18	−7	−86	−3	9.67	<0.000001	7799

All x, y, and z values given here represent stereotaxic coordinates according to the coordinate system by Talairach and Tournoux (1988). The statistical values in the next columns correspond to the *t*-statistics and the *p*-values of the activation maxima (peak voxel) within each anatomical region. All activations were thresholded at q(FDR) <0.05 (t = 3.71). *(BA = Brodmann area; R = right hemisphere; L = left hemisphere)*.

**Table 4:** List of fMRI results for the contrast Block-Ending > Block-Beginning.

Anatomical Region	BA	x	y	z	t - statistic	p - value	No. of voxels
*(Block-Ending > Block-Beginning)*							
/medial frontal gyrus	9	−1	40	27	3.95	0.001252	164
R middle frontal gyrus	9/46	44	31	21	5.38	0.000074	754
L middle frontal gyrus	9/46	−45	25	24	4.76	0.000255	945
R insula	13	29	24	9	5.96	0.000026	651
L & R supplementary motor area	6	−1	10	57	5.8	0.000035	3245
/anterior cingulate gyrus	24	−1	−5	27	5.7	0.000042	832
L & R thalamus (anterior nucleus)		−7	−17	15	5.22	0.000104	902
R superior temporal gyrus	22	47	−29	0	5.38	0.000075	203
R inferior parietal lobule	40	56	−32	27	6.07	0.000022	1423
L inferior parietal lobule	40	−57	−34	27	6.8	0.000006	904
L superior temporal gyrus	22	−64	−35	9	6.65	0.000008	1614
L precuneus/superior parietal lobule	7	−6	−47	45	6.12	0.000020	1396
R precuneus/superior parietal lobule	7	29	−50	48	5.71	0.000042	3311
L & R inferior occipital/lingual gyrus (extending towards BA 37)	18/19/37	−7	−77	6	11.48	<0.000001	>9999

All x, y, and z values represent stereotaxic coordinates according to the coordinate system by Talairach and Tournoux (1988). The statistical values in the next columns correspond to the *t*-statistics and the *p*-values of the activation maxima (peak voxel) within each anatomical region. All activations were thresholded at q(FDR) <0.05 (t = 3.71). *(BA = Brodmann area; R = right hemisphere; L = left hemisphere)*.

**Figure 7:**
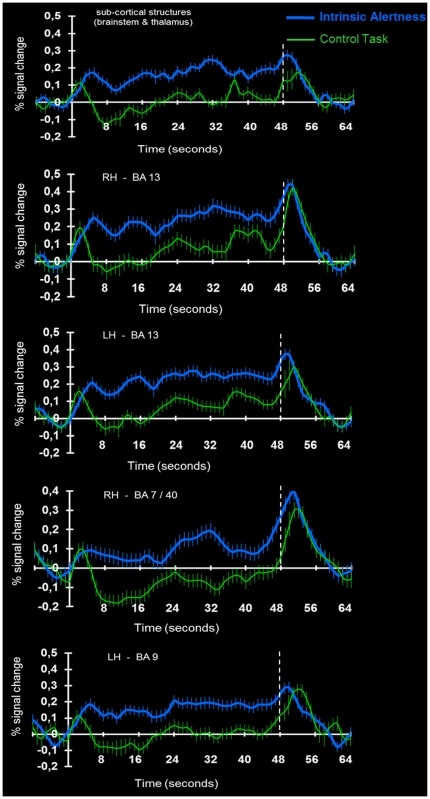
Averaged time course plots of the BOLD response during intrinsic alertness- and control task. Averaged time course plots of the BOLD response during the intrinsic alertness task and the control task, relative to the low-level baseline condition (fixation). For several clusters of interest, including sub-cortical and cortical structures, averaged time course plots illustrate the BOLD response, averaged across 16 participants, during both the intrinsic alertness task (blue line) and the control task (green line). Relative to the low-level baseline condition, the averaged time course plots depict the amount of signal change for both the intrinsic alertness- and the control task, during the whole experimental run. Error bars represent the SEM, averaged across all participants, and the dashed white line marks the end of the block. Time zero always corresponds to the beginning of the experimental block. *(BA = Brodmann area; RH = right hemisphere; LH = left hemisphere)*.

In order to visualize – across the whole experimental run – that the BOLD signal during the intrinsic alertness task was consistently higher as compared to the BOLD signal during the control task, we created averaged time course plots of brain activity for both tasks separately. Figure 7 shows averaged time course plots for several clusters of interest, including cortical and sub-cortical structures. As illustrated in Figure 7, the BOLD response during the intrinsic alertness task, when compared to the control task, was increased in all brain areas of interest for the complete duration of the experimental run. Thus, the task-related differences reported here for the contrast Intrinsic Alertness Task > Control Task represent greater activation during the intrinsic alertness task than during the control task. We take this as further evidence that the level of intrinsic alertness was higher during the experimental task, as compared to the control task. A strong increase in the BOLD response, which is evident in all plots shown, can also be observed after the end of the task block.

**Figure 8:**
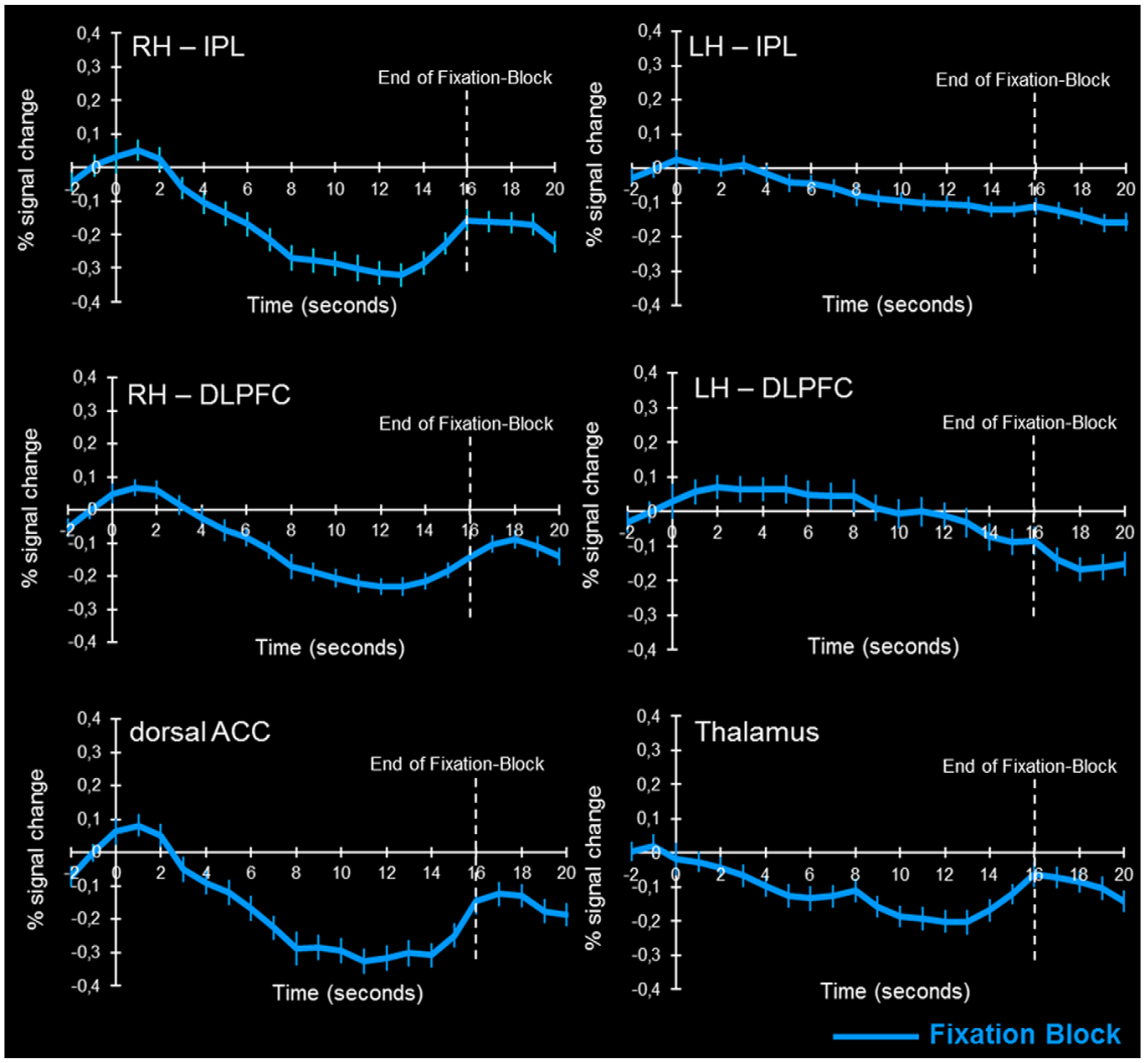
Averaged time course plots of the BOLD response during the low-level baseline condition (fixation). For several clusters of interest, averaged time course plots illustrate the evolution of the BOLD response, averaged across all 16 participants, during the fixation block (blue line). Relative to the last 3 time points (4.8 s) of the intrinsic alertness task, the averaged time course plots depict the amount of signal change for the complete duration (16 s) of the fixation block. Error bars represent the SEM, averaged across all participants, and the dashed white line marks the end of the fixation block. Time zero always corresponds to the beginning of the fixation block. *(ACC = anterior cingulate cortex; DLPFC = dorsolateral prefrontal cortex; IPL = inferior parietal lobule; RH = right hemisphere; LH = left hemisphere)*.

During the second part of the analysis, averaged time course plots explicitly showing the evolution of the BOLD response during the low-level baseline condition (fixation) were calculated, in order to capture any internal up-regulation of arousal possibly happening before the intrinsic alertness task actually started. Thereby we aimed to verify whether the change of the screen color accompanying the change of the fixation and task conditions was effective in preventing increased activity within intrinsic alertness related brain areas already during the fixation block. Figure 8 depicts these plots for some of the crucial areas of the intrinsic alertness network (right IPL and right DLPFC, dorsal ACC and thalamus), and for two other brain areas (left IPL and left DLPFC) not typically involved in intrinsic alertness. While increased BOLD responses shortly before the start of the task block could be expected in intrinsic alertness-related brain areas, due to enhanced expectancy shortly before the switch to the task block, we would not expect such effects in the left parietal and frontal areas since they should not be directly related to the internal regulation of arousal due to expectancies concerning the start of the next task block. A corresponding pattern of results can be seen in Figure 8, showing all averaged time course plots for the low-level baseline condition. There was an increase in the BOLD signal clearly before the end of the fixation block in all intrinsic alertness-related areas, but not in the left IPL and DLPFC. In those two areas, activity continued to decrease throughout the fixation period, and there was no increase in the BOLD response before the end of the fixation block.

## Discussion

### 4.1 Overall results

With the present study we sought to further elucidate the functional neuroanatomy of intrinsic alertness, and the primary goal was to map the neural correlates of this attentional function as specifically as possible. We examined intrinsic alertness in healthy participants, using well-controlled and standardized experimental paradigms combined with hypothesis-driven statistical analysis. The experimental paradigms were explicitly optimized to provide a specific evaluation of those brain areas involved in the processing of intrinsic alertness. Behavioral results of the present study indicated that all participants executed the intrinsic alertness task with good accuracy. This indicates a normal level of task performance across all participants. Furthermore, the behavioral analysis revealed that the number of key presses was significantly higher under the control condition. Nevertheless, we found higher activation of the SMA under the alertness condition. This result seems to corroborate the general conceptualization that the SMA is crucially involved in the planning of controlled motor responses to external stimuli. Thus, the higher SMA activation under the alertness task could be explained by a higher top-down demand on motor control, in contrast to the more automatized, repetitive motor actions in the control task.

The fMRI results of the present study further revealed activation of a fronto-parietal-cingulate-thalamic-brainstem network, as similarly postulated for intrinsic alertness by previous neuroimaging studies [12], [14]–[16], [27]–[30], [34]. However, a clear right-lateralization of the present results was not found, since several clusters of activation were located within the left hemisphere, and right BA 9 activity was only revealed by more fine grained analyses examining specifically the last time period of the intrinsic alertness task. Especially these more fine grained analyses revealed that several areas of the brain seem to be differentially involved in the intrinsic alertness task, depending on whether the task has just started or is about to end. Based on our findings, we suggest that maybe the most consistent feature of the functional neuroanatomy of intrinsic alertness might be activation of the noradrenergic ascending system (brainstem, thalamic and cingulate areas). Overall, our results are in accordance with previous results from the literature. But they also revealed that the functional neuroanatomy of intrinsic alertness, if assessed with a block design in fMRI studies, seems to be less right-lateralized, possibly due to an involvement of phasic alertness triggered by the switch between task and rest conditions.

### 4.2 Involvement of brainstem-thalamic-cingulate structures during intrinsic alertness

As proposed in one of our former studies [23], we suggest that the ACG serves as some sort of regulating or integrating component of the intrinsic alertness network. This conclusion is supported partly by the fact that this part of the brain showed the strongest and spatially most extended BOLD response, except for V1 and V2, during the intrinsic alertness task. These strong BOLD responses within primary and secondary visual cortices could be related to processing of intrinsic alertness. But on the other hand the results of the present study do not allow us to exclude the possibility that an increase of visual activations during the intrinsic alertness task might not be due to an enhanced alerting state but possibly a consequence of differences in the visual stimulus material used. While the intrinsic alertness task entails a dynamic display of visual stimuli, the control task just shows a static image, and one could argue that dynamic visual displays lead to a stronger activation of the visual cortices. On the other hand, the actual dynamics of the stimuli of the intrinsic alertness task were rather ‘reduced’, and the long-lasting presence of the stimuli during the control task may also represent a strong visual stimulation in itself. However, the activation differences within visual cortices were not the primary focus of the present study. Besides these strong visual activations, the second strongest activation was that of the ACG, being the only brain area which showed increased activation in all contrasts from both parts of the analysis. The ACG and the surrounding anterior cingulate cortex (ACC) are well known for integrating and coordinating anticipatory and preparatory attentional activity [48], [49], because this area of the brain is densely and reciprocally connected to the noradrenergic and cholinergic subcortical systems responsible for the cognitive control of arousal [50]–[53]. Several other functions, such as motor control, homeostatic drive, self-regulation and general cognitive control, have also been assigned to the ACG [54]–[56]. But based primarily on previous findings [23], we cautiously suggest that the ACG serves as the central regulating component within the neural network for intrinsic alertness, responsible for task-dependent modulation of arousal: according to the task demands during the processing of intrinsic alertness, the ACG seems to modulate and control the subcortical structures providing noradrenergic bottom-up activity [11], [23], [35], [57]. Nevertheless, it has to be acknowledged that the results of the present study alone do not allow for an unambiguous definition of the function of the ACG during intrinsic alertness.

The brainstem activation in the present study could represent the anatomical origin of the noradrenergic activation necessary for the processing of intrinsic alertness, and this interpretation is in line with several previous studies evaluating intrinsic alertness [16], [27], [30], [57]. However, it should be noted that the coarse spatial resolution of fMRI actually does not allow drawing direct and reliable conclusions on whether specific brainstem structures, such as the locus coeruleus as the origin of the noradrenergic bottom-up system [50], were truly activated during intrinsic alertness. The thalamic activation in the present study also fits with results from previous studies, since the thalamus most likely functions as some sort of gating system, diverting ascending noradrenergic activation towards different cortical structures [27], [58], [59]. Thus, the primary function of the thalamus during intrinsic alertness might be to relay sub-cortical bottom-up activity towards cortical structures, such as the ACG, and simultaneously to relay top-down signals from cortical areas back to brainstem structures such as the locus coeruleus. Such an interactive view on bottom-up and top-down processes during intrinsic alertness seems to make sense, because only a dynamic interplay between cortical and sub-cortical structures can provide a flexible control mechanism to cognitively regulate arousal. All of the above could be taken to corroborate the hypothesis of a top-down controlled noradrenergic system representing intrinsic alertness [11], [24], [25]. Thus, we cautiously suggest that the brainstem-thalamic-cingulate part of the functional neuroanatomy of intrinsic alertness represents a crucial component of the neuroanatomy of this attentional function.

### 4.3 The role of parietal and insula activations for intrinsic alertness

Although the intrinsic alertness task did not involve re-orienting or spatial shifts of attention, the right parietal cortex (BA 7/40) also showed significant activation. These parietal activations can be interpreted as a co-activation of the attentional system responsible for spatial orienting of attention [16], [24], [25], [30], or as representing an inherent component of the stimulus-driven attention system [13], [14]. Although this question cannot be completely resolved here, we would favor the latter conclusion, since activation of right parietal cortex seems to be a robust finding in neuroimaging studies on basal aspects of attentional processing [4]. Right parietal cortex was active in the contrast Intrinsic Alertness Task > Control Task, and also in the contrasts specifically looking for patterns of activations at the end of each task block in the second part of the analysis. We suggest that the general function of the right parietal cortex during intrinsic alertness is related to the processing of information about the behavioral relevance and salience of stimuli. This information can subsequently be used to interrupt ongoing cognitive activity in case of unexpected or novel stimuli. Thus, during intrinsic alertness, right parietal cortex most likely processes bottom-up information combined with predictive information in order to facilitate rapid target detection [13].

Bilateral activation of the anterior part of the insula could be related to some attentional integrating function, which is one of the multiple functions postulated for this part of the brain and the surrounding inferior frontal junction (IFJ), consisting of adjacent parts of BA 9, 13, 44, and 45 [2]. Previous studies, using auditory stimulus material to assess intrinsic alertness, interpreted activations within this part of the brain (BA 13/45) as representing the frontal regulating component of a fronto-temporo-parietal alertness network [30]. Asplund and colleagues recently suggested that the IFJ in particular and the lateral portion of the prefrontal cortex in general, sub-serve some sort of attentional interaction between stimulus-driven and goal-directed attention [2]. This conclusion could also apply to the present results, because the task requirements during the present experiment inevitably involve some sort of more complex, goal-directed attentional processing. This goal-directed attentional processing at some point has to interact with stimulus-driven attention, in order to produce a coherent behavioral response [2], [13]. The anterior part of the insula in particular, or the IFJ in general, could represent the locus within the brain where this interaction is processed. As noted elsewhere [2], such an attentional interaction between bottom-up and top-down processing seems to be inevitably involved in any task demanding attentional processing and a corresponding behavioral response.

### 4.4 Interpretation of left frontal activations during intrinsic alertness, assessed with fMRI block designs

Interestingly, the left frontal activations reported here are quite comparable, concerning spatial layout and localization, with the left-hemispheric activations for phasic alertness mentioned earlier [30]–[34]. To account for this complex pattern of results, and more specifically for the activations within left BA 9 and the absence of right BA 9 activity during intrinsic alertness, we propose the following explanation: it cannot be excluded, that the block design of the experiment used to assess intrinsic alertness also involved processing of phasic alertness to some degree. Especially the change of the screen color between the fixation and task conditions may have unintentionally served as an external (warning) cue indicating when alertness needed to be increased and when the appearance of the first target stimulus was to be expected. This could have contributed to an activation of the phasic alertness system. Generally, it is assumed that the presentation of an external cue before the target stimulus in alertness task recruits additional left fronto-parietal brain areas, resulting in a more bilateral activation pattern as compared to intrinsic alertness [5], [30]. Furthermore, the only additional brain areas associated with the functional neuroanatomy of phasic alertness, as compared to the functional neuroanatomy of intrinsic alertness, are located within left prefrontal cortex [6], [16], [35], [60]. Therefore, an involvement of phasic alertness during the experimental task may partly explain the left frontal activations reported here.

Such a rather complex pattern of results might emerge partly because researchers always need to induce the state of intrinsic alertness in an artificial manner, in order to assess it with fMRI. Most likely, the crucial network for intrinsic alertness is truly lateralized to the right hemisphere, as nicely illustrated by numerous lesion studies [17]–[21] and various neuroimaging studies [12], [14]–[16], [28], [29]. But due to the repeated switching between rest and task conditions involved in block designs used to assess intrinsic alertness in fMRI or PET studies, co-activation of the phasic alertness system can hardly be prevented completely, which in turn results in less lateralized activation patterns. In other words, left-frontal activity and the absence of right-frontal activity could be viewed as a sort of methodological artifact, because the changing screen colors and the temporally identical intervals between task and fixation periods inevitably and unintentionally provide some sort of external cueing for the participant. Participants then most likely use these external cues to increase or decrease alertness (i.e. phasic alertness), rather than relying solely on internal up-regulation of arousal (i.e. intrinsic alertness). Such a more externally cued processing of alertness would probably lead to a stronger BOLD response in the left DLPFC, which is more responsible for phasic alertness, than in the right DLPFC, which is more responsible for intrinsic alertness [5], [11]. This rationale provides a methodologically motivated explanation for the absence of right DLPFC activity and the presence of left DLPFC activity during intrinsic alertness in the present study. The strongly increased BOLD response after the task block which was evident for all averaged time course plots from the first part of the analysis (see Figure 7), was probably much less internally triggered but more externally induced by an attention-capturing event such as the changing screen color. This implies that the increased BOLD response immediately after the end of the task block could represents a sort of ‘phasic offset response’, which would be assigned primarily to processing of phasic (externally modulated) and not intrinsic (internally modulated) alertness. We interpret this as further support for the hypothesis that due to the repeated switching between fixation and task conditions involved in block designs used to assess intrinsic alertness, a co-activation of the phasic alertness system and additional left-frontal activity can hardly be prevented.

Thus, the block design itself might have a profound influence on how the brain processes the intrinsic alertness task, and further support for this hypothesis comes from the results of the contrasts of the second part of the analysis. The fact that very different results were found when looking at the beginning and the end of the task block implies that some external cueing (e.g. change in screen color) triggered the beginning of each task block for the participants. This reduced the need for intrinsic alertness and internal up-regulation of arousal because participants could more efficiently rely on external cueing. The widespread presence of less activation, relative to the low-level baseline, at the beginning of the block could be interpreted in so far, as that the participants did not need a lot of activity in alertness related brain areas to perform the beginning of the task. This was probably due to the simple nature and the previous rehearsal of the task, and due to the expectancies of the participants about when the next task block and the next stimulus appears. This together, external cueing and gross estimation of temporal intervals could reduce the need to activate any alertness network in the brain, resulting in no or less activation, relative to the low-level baseline, of the brain. Thus, the different activation patterns at the beginning and the end of the block can most likely be attributed to methodological peculiarities of the block design used to assess intrinsic alertness. Furthermore, contrasting any predictor with the fixation condition could wipe out right frontal activations to some degree because these are also present at the end of the fixation phase, due to enhanced expectation and alertness processing induced by the screen change. What might seem contradictory at first is that there is no clear right-lateralization in the Block-Ending > Block-Beginning contrast. If a phasic cueing effect influenced brain activity, this effect should decline over time and theoretically be weakest towards the end of the task block. However, the predictors for the Block-Ending and Block-Beginning encompassed multiple TR's and did not cover specifically only the end and the beginning of the blocks, which might lead to the more bilateral results in the Block-Ending > Block-Beginning contrast. Overall, a clear-cut right-lateralized activation pattern might be hard to detect because phasic cueing effects could be present to a varying degree throughout the whole task block. This reduces the necessity for the brain to employ only intrinsic alertness to solve the task, which in turn strongly reduces the probability to find strongly right-lateralized fronto-parietal activations at any point in time during the experimental task. Even in earlier PET studies on intrinsic alertness besides strong right there always also was weaker left hemisphere activity [16], [30].

The fact that temporal expectancies and estimation of intervals might be used by the participants to optimize task performance, even without any explicit task instruction to do so, implicates that temporal expectations could have influenced the present results at least to some degree. If participants used implicit timing, defined as a temporal estimation engaged by temporally structured sensory information [61], this could have contributed to the observed left frontal, premotor, and cerebellar activations. Recent fMRI studies suggest that implicit timing and temporal expectations either directly influence sensory (V1/V2), motor (SMA), and reward-related prefrontal areas (DLPFC), or indirectly influence ongoing processing in those areas [61]–[64]. Most consistently, left premotor and inferior parietal areas have been activated during implicit timing, and a left-hemispheric preference for implicit timing has been suggested by different studies [61], [65]. Based on these previous results [61], [62], [65], because we also found left-frontal activations not normally observed during intrinsic alertness, and because our design contained temporally structured sensory information that could be used to optimize task performance, we suggest that left-hemispheric sensory, motor and prefrontal activations could be influenced by temporal expectations. However, due to the fact that we had no measure of the degree to which our participants used implicit timing or not, and because the set of brain areas specifically processing implicit timing is not yet defined, it seems problematic to determine the exact influence that this factor had on the present fMRI results.

A challenge for future studies seems to be to possibly find a reliable way to assess intrinsic alertness using fMRI, without evoking activity within the phasic alertness network at the same time and without employing temporal expectations. A block design might not provide the optimal solution for this challenge, because due to expectancies or gross estimation of temporal intervals during the experiment, participants could have been able to increase their level of alertness even before the task started, as an internal preparation for the beginning of the next task-block. This relates to one of our general concerns with fMRI studies using block designs: activation within the low-level baseline condition, which is inevitably present in any block design to allow the BOLD response to return to baseline, is subtracted from the activation during the task condition. And this might also happen when the activation in brain areas of interest is already increased during the low-level baseline condition. Such a rationale also provides an explanation for the results of Figure 4. One would expect to find left-hemispheric phasic alertness activity in the SC 1 > Fixation contrast, and a more bilateral activation pattern for the SC 2 > Fixation contrast, because the phasic cueing effect should decline over time. Figure 4 shows that the pattern of activity is more bilateral in the SC 2 > Fixation contrast, which could be due to the phasic cueing effect diminishing over time and becoming weakest at the end of the task block. Therefore, the SC 2 predictor shows activation within a distributed network of brain areas, revealing an involvement of both intrinsic and phasic alertness-related brain areas. But why don't we see more lateralized left-hemsipheric activations in the SC 1 > Fixation contrast? Even though the phasic cueing effect might be strongest at block-onset, phasic alertness activity cannot be depicted in Figure 4 because the preliminary increase of activation in alertness-relevant brain areas during the end of the low-level baseline condition is later subtracted from the activations immediately after the end of the low-level baseline (SC 1 predictor). When contrasting fixation and the SC 1 predictor, right-frontal activations might be cancelled out because they are also present in the fixation condition to some degree: if the activation level in relevant brain areas was already quite high during the end of the fixation condition, and if these high activations are later subtracted from the activations immediately after the end of the fixation period (SC 1 predictor), this could lead to the observed results for the SC 1 activation map.

A preliminary increase in brain activity can be seen in Figure 8, where only the averaged time course plots for intrinsic alertness-related brain areas show increased BOLD responses before the task block starts. The clearly visible absence of such a preliminary increase in the BOLD response for the left IPL and DLPFC further implies that those areas are not subserving intrinsic alertness to the same degree. The authors of course have to admit that this is not the only possible explanation for the preliminary increase in BOLD activity. An alternative explanation might be that the observed increase in activation towards the end of the fixation period simply represents a stronger return from post-stimulus BOLD undershoot to baseline. This post-stimulus return to baseline might resemble increasing activation and could be stronger in some brain areas than in others. However, it has to be acknowledged that a preliminary increase in BOLD activity, or a stronger return from post-stimulus BOLD undershoot to baseline, for the left IPL and DLPFC is clearly absent, and not just weaker as compared to the right IPL and DLPFC. Thus, with the present fMRI study, we found increased BOLD responses due to internal up-regulation of arousal for all crucial areas of the intrinsic alertness network, but unexpectedly shortly before the end of the fixation block. The expectancies of the participants about when the next task block starts, which are inevitably tied to any block design with previous task rehearsal, seem to have specifically induced activation in the right hemisphere network for intrinsic alertness towards the end of the fixation block. Overall, this indicates that a block design might unintentionally induce intrinsic alertness processing during fixation periods, and trigger additional phasic alertness processing because participants could employ external cueing to increase or decrease alertness rather than relying on internally regulated arousal. Thus, we cautiously suggest that left-frontal activations, constituting a key feature of the functional neuroanatomy of phasic alertness, observed during the intrinsic alertness task are most likely attributable to the method (fMRI block design) used to assess intrinsic alertness.

Increased activation in intrinsic alertness related brain areas already during the fixation period could have further contributed to the presence of left BA 9 and the absence of right BA 9 activity, because any potential activation of the intrinsic alertness network during the fixation block will be averaged over the 8 fixation blocks and then subtracted from the activations during the intrinsic alertness task. Thus, it is more likely that only left frontal activations remain, because for example right frontal activations might have simply been cancelled out due to the subtraction logic used to construct the Intrinsic Alertness Task > Control Task contrast. Within the present study we found that only the intrinsic alertness related brain areas showed increased BOLD responses during the fixation block, as indicated by Figure 8. These responses during the fixation block were most likely caused by a combination of gross estimation of temporal intervals and approximate expectancies about when the next task block starts (implicit timing), causing participants to internally up-regulate alertness before the end of the fixation block in order to prepare for the start of the next task block. So on the one hand, left frontal activations may remain in the Intrinsic Alertness Task > Control Task contrast because of the external cueing effect (i.e. changing screen color) which triggered the phasic alertness network, of which the left BA 9 is a key component. And in addition to this, increased activation within the intrinsic alertness network could not show up in the Intrinsic Alertness Task > Control Task contrast because of the subtraction logic used to construct this contrast. Since the presence of left BA 9 and the absence of right BA 9 activity were caused predominately by the mere presence of the fixation block, which is inevitably tied to any block design, we interpret these findings as a methodological peculiarity related to the fMRI block design itself. The complex pattern of results reported here could also be taken to indicate that attentional networks are very hard, if not impossible, to functionally separate in block design fMRI experiments. Since nearly all attentional accounts have acknowledged the need for different attentional functions to interact in order to produce coherent behavioral responses [2], [5], [13], it seems logical that some sort of attentional interaction will always influence neuroimaging research of attention. The fact that the coordinates of the BA 13 activations of the present study are almost identical to those reported in [2] raises the question whether the BA 13 activations of the present study can be specifically assigned to intrinsic alertness. But one conclusion of the present study is that as long as a coherent behavioral response from the participant is needed for the assessment of intrinsic alertness in fMRI block designs, any interpretation of the results of such studies should try to reflect the critical points mentioned above.

To conclude, we have partially replicated and extended previous findings concerning the functional neuroanatomy of intrinsic alertness. Using a block design and standardized assessment paradigms from a neuropsychological test battery, we were able to replicate previous findings and found a fronto-parietal-cingulate-thalamic-brainstem network active during intrinsic alertness. Compared to previous results from the literature, we found a more bilateral neural network in the present study. In addition to commonly reported functional activations during intrinsic alertness, we found activation of the anterior part of the insula (bilateral) and the left BA 9 during the intrinsic alertness task. The present results indicate that the frontal and parietal brain areas within the right hemisphere, in addition to brainstem-thalamic-cingulate structures, represent crucial components of the functional neuroanatomy of intrinsic alertness. fMRI experiments in general, and block designs in particular, however seem not to be perfectly well suited to isolate these right hemisphere components and at the same time this type of experimental design seems to provoke the activation of additional brain areas within the left hemisphere. In the light of the present findings we must conclude, that the assessment of intrinsic alertness with fMRI, using block designs, can lead to the co-activation of the phasic alertness network because the repeated switch between task and rest conditions can be taken as a warning cue indicating when the level of alertness must be increased or decreased. Additionally, the second part of our analysis implies that – contrary to our initial hypothesis – the block design itself might induce processing of intrinsic alertness already during fixation conditions, due to expectancies or gross estimation of temporal intervals using implicit timing expectations. This methodological factor has further consequences because the difference between the activation levels for the task block and the fixation block, for example within the right BA 9, will be much smaller as compared to other brain areas such as left BA 9. According to the logic of the RFX GLM, this will greatly reduce the probability for right BA 9 to be activated in the Intrinsic Alertness Task > Control Task contrast, and this in turn may have strongly contributed to the overall presence of left BA 9 and the absence of right BA 9 activity in the present study.

All of these considerations indicate that the assessment methodology itself (fMRI block designs) might induce processing of intrinsic or phasic alertness, irrespectively and sometimes independently of the task the participants are executing. Thus, while block designs might be generally suitable to reveal the functional neuroanatomy of intrinsic alertness, including activation of thalamic-brainstem-cingulate areas in the present study, they might also produce some methodological artifacts, such as left-frontal activations, which are most likely not part of the functional neuroanatomy of intrinsic alertness. The interpretation presented here, that left-frontal activations in the present study are predominately related to an activation of the externally cued phasic alertness network fits with the previous results from the literature: the presentation of an external cue before the target stimulus in alertness tasks seems to recruit additional left fronto-parietal brain areas, resulting in a more bilateral activation pattern as compared to intrinsic alertness [5], [30]. Methodological factors related to the assessment methodology (fMRI block design) might play an even greater role when studying the specific neural correlates of attentional functions such as intrinsic alertness, which are known to possess a high degree of cognitive and neuronal overlap with other attentional functions, such as phasic alertness. The present findings thus further contribute to understanding attentional processing in the human brain, because the research presented here tackled one of the small remaining inconsistencies within the neuroimaging literature on attentional processing [4]. We revealed two methodological peculiarities for fMRI studies investigating intrinsic alertness: first, the repeated change between rest and task blocks may induce external cueing and thus trigger activation within the phasic alertness network, and secondly, expectancies and approximately estimating the start of the next task block may induce intrinsic alertness processing during fixation blocks, and this activity is in turn subtracted from the activations during the intrinsic alertness task. We suggest that both processes might interact and contribute rather strongly to the presence of left BA 9 and the absence of right BA 9 activity in the present study.
