# Correction: Revealing the Functional Neuroanatomy of Intrinsic Alertness Using fMRI: Methodological Peculiarities

**DOI:** 10.1371/annotation/71f1cf97-9ef2-4fa5-8b01-6e3cc05210bb

**Published:** 2013-09-23

**Authors:** Benjamin Clemens, Mikhail Zvyagintsev, Alexander T. Sack, Armin Heinecke, Klaus Willmes, Walter Sturm

The name of the third author was given incorrectly. The correct name is: Alexander T. Sack. 

The correct citation is: Clemens B, Zvyagintsev M, Sack AT, Heinecke A, Willmes K, et al. (2011) Revealing the Functional Neuroanatomy of Intrinsic Alertness Using fMRI: Methodological Peculiarities. PLoS ONE 6(9): e25453. doi:10.1371/journal.pone.0025453. 

The correct abbreviation in the Author Contributions statement is: ATS. 

